# Boosting *In Planta* Production of Antigens Derived from the Porcine Reproductive and Respiratory Syndrome Virus (PRRSV) and Subsequent Evaluation of Their Immunogenicity

**DOI:** 10.1371/journal.pone.0091386

**Published:** 2014-03-10

**Authors:** Robin Piron, Stefaan De Koker, Annelies De Paepe, Julie Goossens, Johan Grooten, Hans Nauwynck, Ann Depicker

**Affiliations:** 1 Department of Plant Systems Biology, VIB, Ghent, Belgium; 2 Department of Plant Biotechnology and Bioinformatics, Ghent University, Ghent, Belgium; 3 Department of Biomedical Molecular Biology, Ghent University, Ghent, Belgium; 4 Department of Bioscience Engineering, VUB, Brussels, Belgium; 5 Department of Virology, Parasitology and Immunology, Ghent University, Ghent, Belgium; Ghent University, Belgium

## Abstract

Porcine reproductive and respiratory syndrome (PRRS) is a disease of swine, caused by an arterivirus, the PRRS virus (PRRSV). This virus infects pigs worldwide and causes huge economic losses. Due to genetic drift, current vaccines are losing their power. Adaptable vaccines could provide a solution to this problem. This study aims at producing *in planta* a set of antigens derived from the PRRSV glycoproteins (GPs) to be included in a subunit vaccine. We selected the GP3, GP4 and GP5 and optimized these for production in an *Arabidopsis* seed platform by removing transmembrane domains (Tm) and/or adding stabilizing protein domains, such as the green fluorescent protein (GFP) and immunoglobulin (IgG) ‘Fragment crystallizable’ (Fc) chains. Accumulation of the GPs with and without Tm was low, reaching no more than 0.10% of total soluble protein (TSP) in homozygous seed. However, addition of stabilizing domains boosted accumulation up to a maximum of 2.74% of TSP when GFP was used, and albeit less effectively, also the Fc chains of the porcine IgG3 and murine IgG2a increased antigen accumulation, to 0.96% and 1.81% of TSP respectively, while the murine IgG3 Fc chain did not. Antigens with Tm were less susceptible to these manipulations to increase yield. All antigens were produced in the endoplasmic reticulum and accordingly, they carried high-mannose N-glycans. The immunogenicity of several of those antigens was assessed and we show that vaccination with purified antigens did elicit the production of antibodies with virus neutralizing activity in mice but not in pigs.

## Introduction

PRRSV is a pig pathogen that emerged in the late eighties in North America and North Europe [1], [2]. Since its emergence, PRRSV has spread across the globe. It is considered one of the major pathogens affecting pig industries and causes severe economic losses worldwide [3], [4]. It provokes respiratory distress in pigs of all ages, but is especially problematic when infecting pregnant sows, leading to late abortion, early farrowing and birth of dead or weakened piglets [5]. Lately, more virulent strains with a high incidence of pig mortality have been circulating [4], [6].

PRRSV is an enveloped RNA virus belonging to the family of the *Arteriviridae*. Two genotypes, a North American (NA-type) and a European (EU-type), have been identified, which again can be divided in several subtypes [7], [8]. It has multiple open reading frames embedded in its 15-kb long genome [9]. Open reading frames 2 to 7 code for the glycoprotein (GP) 2, the E protein, GP3, GP4, GP5, the M protein and the nucleocapsid protein N, respectively [9]–[12]. Of these structural proteins, the GP5 and the M protein are the major proteins found in the lipid envelope and occur as a disulfide-linked heterodimer [13]. The minor envelope proteins, i.e. GP2, GP3, GP4 and the E protein, are present as non-covalently associated heteromultimers.

PRRSV infection induces a defective immune response with late appearance of neutralizing antibodies and delayed cell mediated immunity [14], [15]. This allows the infection to be persistent and makes it difficult to eliminate PRRSV from an infected herd. Control of PRRSV is focused on the administration of prophylactic vaccines to minimize the clinical impact of an infection. The vaccines currently used in the field consist of killed and live, attenuated vaccines. Commercial killed vaccines are safe and can provide some protection against homologous viruses, but are totally ineffective against heterologous strains [16], [17]. The available attenuated vaccines, however, are very effective against homologous challenges and can, to a certain degree, even cross protect against heterologous strains, but they raise safety concerns because of their live viral content [16]–[20]. The attenuated vaccine can revert to virulence and cause infection in the vaccinated herd instead of preventing it. This has been documented for PRRSV on several occasions [19]–[21]. There is thus an urgent need for an adaptable, safe and effective PRRSV vaccine, such as a rationally engineered subunit vaccine. The latter is inherently safe because no virus is present and by incorporating elements known to be important in immunity against PRRSV, could be very effective. To this goal, we selected GP3, GP4 and GP5 of the European prototype PRRSV, the ‘Lelystad Virus’ (LV), to be included in a subunit vaccine. Since viral glycoproteins are complex proteins that cannot be correctly produced in bacteria, we opted to express them in the seed of *Arabidopis thaliana*, a production platform that has previously been used to generate several heterologous proteins to levels as high as 15% of total soluble protein (TSP) [22]–[26]. Production of high-value pharmaceuticals in plants has gained popularity during the last decades, due to intrinsic advantages offered by this production platform, such as flexible scale up, the absence of human or animal pathogens, and the potential for the cheap production and storage of complex proteins [27]–[29]. The technology has reached maturity and several plant made pharmaceuticals are in clinical trials, on the road to the market [30]. Different plant species and tissues have been employed for the production of PRRSV vaccines. Banana leaves [31], potato tubers [32] and tobacco leaves [33], [34] were used to express the GP5 and recombinant variants thereof. The M protein was produced in corn calli [35] and the N protein in soybean seed [36]. Antigen accumulation was relatively low, ranging from 0.011% of TSP for GP5 expressed in tobacco to 0.65% of TSP for the N protein expressed in soybean seed and none of the antigens were purified. However, to overcome economic hurdles during vaccine production, it is essential that the antigens accumulate to high levels and that an efficient purification protocol is in place. With this in mind, we manipulated the GP3, GP4 and GP5 coding sequence to augment their production and to facilitate purification by removing transmembrane domains (Tm), adding affinity tags and/or fusing them to stabilizing protein domains, such as the ‘Fragment crystallizable’ (Fc) chain of immunoglobulins (IgGs) or the green fluorescent protein (GFP), hence giving rise to an entire set of antigen formats derived from the EU-prototype LV. All these different constructs were cloned in a seed-specific expression cassette [37], transformed into *Arabidopsis thaliana* and the resulting transformants screened for antigen content. The antigens were characterized and evaluated for their ease of production and purification. Further, the immunogenicity of the antigens and their potential to raise neutralizing antibodies were investigated by vaccinating mice, and subsequently piglets, with purified antigens.

This paper details the *in planta* production of a set of antigenic proteins derived from the GPs of the EU-prototype LV. We show that the antigens are correctly produced in the seed, accumulate to levels that are economically feasible (1% of TSP or more; [38]) and can be purified by single-step affinity chromatography. Additionally, we assayed the immunogenicity of several antigens in both a murine and porcine model.

## Results

### Antigen expression in *A. thaliana* seed

**Figure 1:**
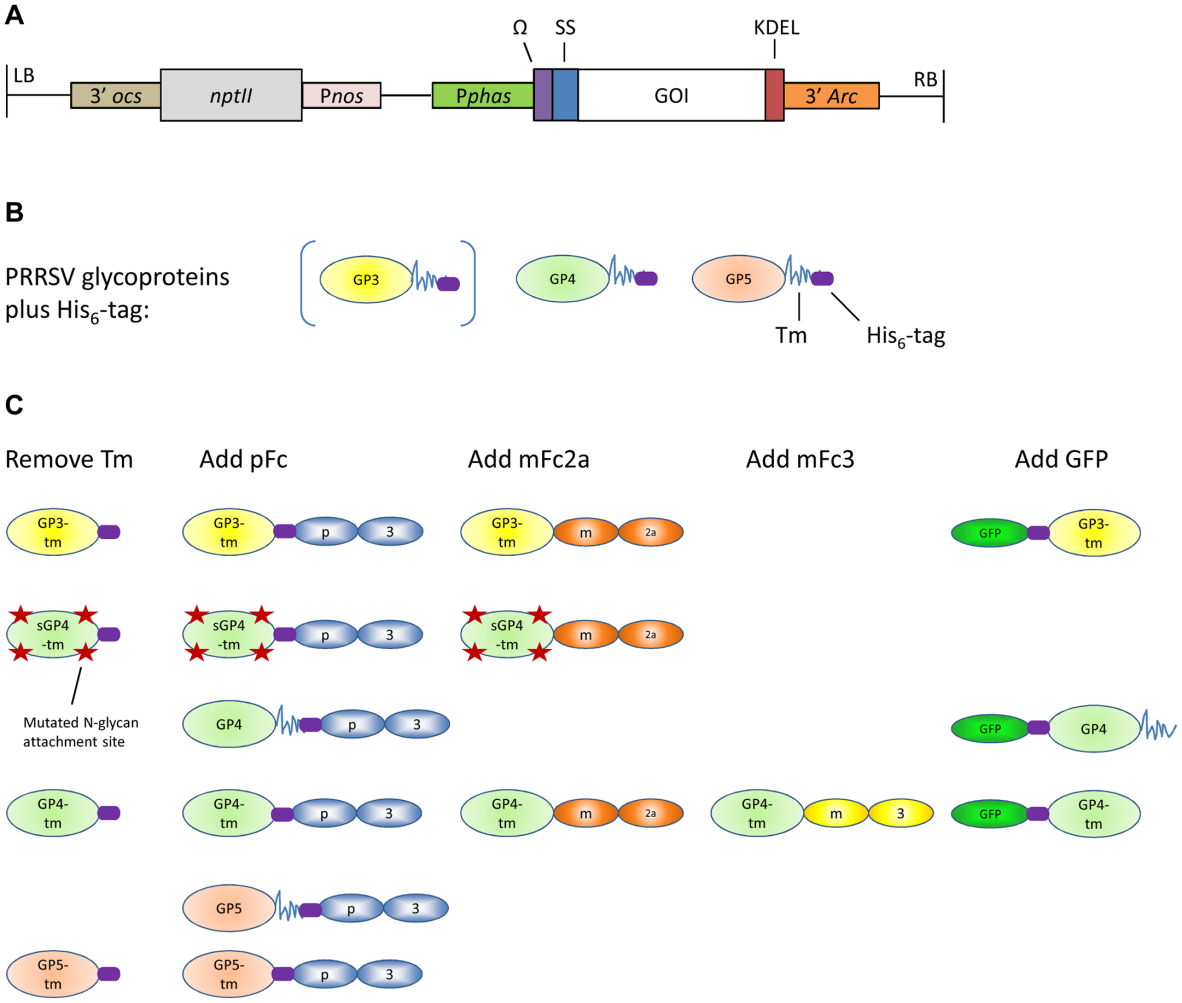
Schematic representation of the T-DNA bearing the seed-specific expression cassette (A), the PRRSV glycoproteins selected to be included in a vaccine (B) and the antigen formats derived thereof (C). (A) The T-DNA carrying the neomycin phosphotransferase II gene which confers resistance to the antibiotic kanamycin and the cassette for high seed-specific expression driven by the *β-phaseolin* promoter, the leader sequence of the tobacco mosaic virus and the *arcelin-5I* terminator. The cassette is further supplemented with sequences encoding an ER-targeting and retention signal. (B) The full-length PRRSV glycoproteins used in the current study and supplemented with a His_6_-tag (purple rod). The jagged line depicts the Tm. The full-length GP3 is placed between brackets as it was never produced as such. Only the GP3 without Tm was expressed, depicted as GP3-Tm in (C). (C) Overview of the manipulations performed on the GPs and the resulting set of antigenic proteins whose coding sequences were cloned into the seed-specific expression cassette. Abbreviations and symbols used: LB and RB, T-DNA left and right border, respectively; 3′*ocs*, 3′end of the octopine synthase gene; *nptII*, the neomycin phosphotransferase II gene; P*nos*, promoter of the nopaline synthase gene; P*phas*, promoter of the *β-phaseolin* gene; Ω, leader sequence of the ‘tobacco mosaic virus’; SS, sequence coding for the signal peptide of the 2S *Arabidopsis* seed storage protein; GOI, gene of interest; KDEL, coding sequence of the ER-retention signal; 3′*Arc*, terminator of the *arceline5-I* gene; Tm, transmembrane domain, jagged line; His_6_-tag, immobilized metal affinity chromatography tag, purple rod; pFc, porcine IgG3 Fc, chain of blue ovals; mFc2a, murine IgG2a Fc, chain of orange ovals; mFc3, murine IgG3 Fc, chain of yellow ovals; GFP, green fluorescent protein, bright green oval.

The PRRSV envelope proteins GP3, GP4 and GP5 (Figure 1B) were selected and expressed as different formats in *A. thaliana* seed (Figure 1C). Both full-length GP4 and GP5, as well as their truncated formats without Tm (hereafter referred to as GP4-Tm and GP5-Tm) were cloned into an expression cassette with regulatory sequences of *Phaseolus vulgaris* designed for high seed-specific expression [22], [24], [37] (Figure 1A). All antigens were targeted to the endoplasmic reticulum (ER) by N-terminal fusion to the signal peptide of a 2S seed storage protein, and retained there via a C-terminal KDEL-tag. Little is known about the transmembrane topology of the full-length GP4 and GP5. The C-terminal hydrophobic domain of GP4 is predicted to span the membrane once, whereas the central hydrophobic domain of GP5 is predicted to span the membrane anywhere from one to three times [10], [39]. *In vivo*, transit through the secretory pathway decorates the GPs with complex mammalian N-glycans [10], [13], [40]. However, retaining the GPs in the ER will cause their N-glycans to be of the high-mannose type [39]. To evaluate the influence of these ER-associated high-mannose glycans on antigen immunogenicity, a deglycosylated format of the GP4-Tm (named sGP4-Tm) was designed. This was done by mutating the asparagine in the consensus sequence N-x-S/T (with x being any amino acid except proline) into a glutamine for each of the four N-glycan attachment sites that was present. For GP3, only a truncated format without Tm (named GP3-Tm) was cloned. Fusions were also made between these proteins and stable protein domains, such as the GFP and the Fc chains of the porcine IgG3 (pFc), the murine IgG3 (mFc3), and the murine IgG2a (mFc2a), generating a whole set of antigenic PRRSV proteins (Figure 1C). All constructs except the murine Fc fusions, contain the His_6_-tag. For every antigen construct, twenty transformants were generated, and the line with the highest antigen content and in which the T-DNA was integrated at a single locus, as determined by segregation analysis (see Material & methods), was retained, selfed and propagated to homozygosity.

**Figure 2:**
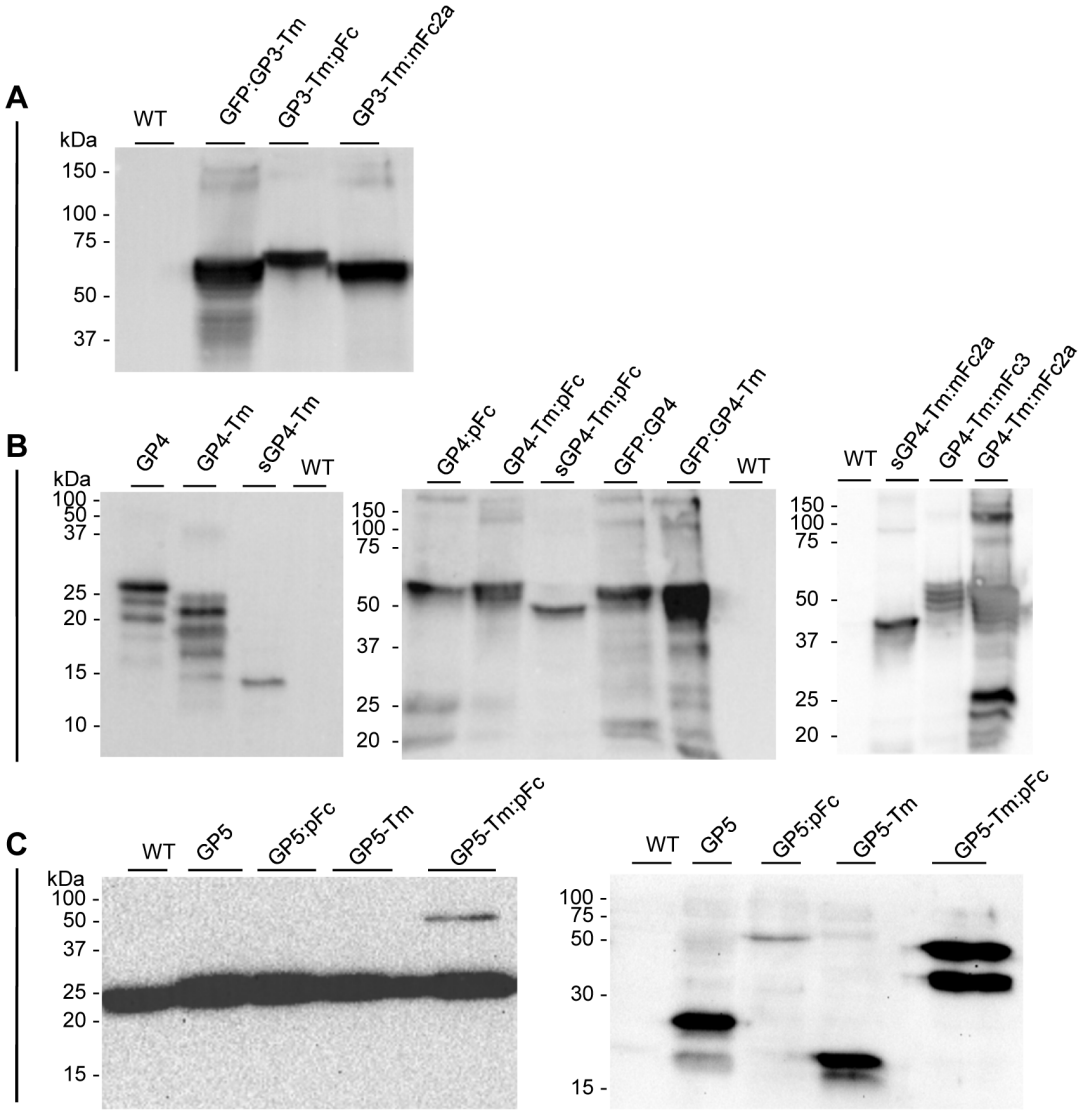
Characterization of the GP3, GP4 and GP5 antigens. Seed extracts of homozygous lines (unless stated differently) producing the different antigen formats were analyzed by western blotting under reducing conditions. Sixty µg of TSP was loaded. (A) The GP3 antigens detected with the α-GP3 mAb VII2D [41]. (B) The GP4 antigens detected with the α-GP4 mAb XVI 11C [42]. For the sGP4-Tm, sGP4-Tm:pFc and the GFP:GP4, segregating T2 stocks were analyzed and for the GFP:GP4-Tm and GP4-Tm:mFc2a, only 15 µg of TSP was loaded. (C) The GP5 antigens detected with the α-GP5 mAb II5A [41] (left panel) or an antibody against the His_6_-tag (right panel). For the GP5-Tm:pFc, a T2 segregating stock was analyzed. Abbreviations used: WT, wild-type seed extract; kDa, kilodalton.

Transformants could be obtained for all GP3 derived constructs, except for GP3-Tm. Extracts from homozygous GP3-Tm:pFc, GP3-Tm:mFc2a and GFP:GP3-Tm seed stocks were analyzed by western blotting with the anti- (α-) GP3 monoclonal antibody (mAb) VII2D that recognizes the linear epitope MWCKIGHDRCEE [41]. This revealed several glycoforms migrating at their expected theoretical molecular weight (Mw) ranging from 46.3 kDa (one of the six or seven N-glycan attachment sites occupied) to 60.3 kDa (fully glycosylated) (Figure 2A; Table 1). Mainly for the GFP:GP3-Tm also some non-reduced multimeric structures could be seen between 100 and 150 kDa, together with some degradation products around 37 kDa.

**Table 1:** Physical parameters of the antigens.

Antigen	Amino acids	N-glycosylation sites	Predicted molecular weight (kDa)	
			Unglycosylated	Fully glycosylated
GP3-Tm	166	6	19.2	31.2
GP3-Tm:pFc	407	7	46.3	60.3
GP3-Tm:mFc2a	392	7	44.6	58.6
GFP:GP3-Tm	410	6	46.6	58.6
GP4	176	4	19.5	27.5
GP4:pFc	417	5	46.7	56.7
GFP:GP4	420	4	46.9	54.9
GP4-Tm	158	4	17.6	25.6
GP4-Tm:pFc	399	5	44.7	54.7
GP4-Tm:mFc3	384	6	42.8	54.8
GP4-Tm:mFc2a	384	5	43.0	53.0
GFP:GP4-Tm	402	4	45.0	53.0
sGP4-Tm	158	0	17.6	17.6
sGP4-Tm:pFc	399	1	44.8	46.8
sGP4-Tm:mFc2a	384	1	43.0	45.0
GP5	179	2	19.9	23.9
GP5:pFc	420	3	47.1	53.1
GP5-Tm	119	2	13.6	17.6
GP5-Tm:pFc	381	3	43.1	49.1

The molecular weight of the fully glycosylated antigens was obtained by taking the weight of the unglycosylated antigens and adding to that the number of potential N-glycosylation sites multiplied by 2 kDa. Abbreviation used: kDa, kilodalton.

The GP4 seed stocks were analyzed with the α-GP4 mAb XVI11C that recognizes the linear epitope GVSAAQEKISFG [42] (Figure 2B). Since GP4 and GP4-Tm carry four N-glycan attachment sites, four different glycoforms could be detected for both, with those of GP4 migrating slower than those of GP4-Tm, due to the presence of the Tm (Figure 2B). However, a differential glycosylation preference was observed, since GP4 showed a higher intensity of its upper band and a very weak signal of the second lowest band, whereas GP4-Tm showed a higher intensity of the second band and equal intensities of the other three glycoforms. The signal of sGP4-Tm corresponded with the lower band of GP4-Tm (Figure 2B), confirming that this represents the unglycosylated GP4-Tm. Mutating the N-glycan sites seemed to have a deleterious effect on the accumulation of sGP4-Tm, as shown by the low intensity of its corresponding band. All fusions of GP4, GP4-Tm and sGP4-Tm with pFc, mFc3, mFc2a and GFP migrated at their expected theoretical Mw (Table 1), but with different signal intensities (Figure 2B), pointing toward differences in antigen accumulation in the seed. For GP4-Tm:mFc2a some non-reduced homodimers were visible around 100 kDa together with degraded fragments at 25 kDa (Figure 2B). To a lesser extent, non-reduced multimers and degradation products could also be observed for some of the other antigens (Figure 2B).

Western blot analysis of seed extracts containing the different GP5 antigens and detected with the α-GP5 mAb II5D (which recognizes the linear epitope DSSTYQYIYNLT [41]), showed only for GP5-Tm:pFc a weak signal at 50 kDa (Figure 2C, left panel). In all samples however, even in the wild-type extract, a strong background band appeared around 20–25 kDa, possibly covering other signals. A similar pattern was observed when the α-GP5 mAbs II5A and VII2H were used to detect the antigens (data not shown, it should however be noted that the mAbs II5D, II5A and VII2H recognize the same linear epitope [41]). Western blot analysis was further performed with an antibody against the His_6_-tag, which was also present in the constructs (Figure 1C) and enabled detection of all GP5 antigens (Figure 2C, right panel). As expected, GP5 migrates slower than GP5-Tm. The smaller, faint bands were hypothesized to represent partially or unglycosylated proteins, since both GP5 and GP5-Tm carry two N-glycosylation sites, and this was confirmed by deglycosylation analyses (see next section). GP5:pFc migrates slower than GP5-Tm:pFc, but is only very faintly detected, indicating low expression. GP5-Tm:pFc on the other hand accumulates notably higher than the other GP5 antigens, but also shows a second band migrating at a size smaller than the theoretical Mw of 49 kDa, which does not correspond with the partially or unglycosylated proteins, but represents a cleavage product (see next section).

In conclusion, all antigens, except the GP3-Tm for which no transformants could be obtained, are present in the seed of the transgenic *A. thaliana* and accumulate to different levels. Also, aside from GP5-Tm:pFc that is partially cleaved, all antigens appear structurally integer and migrate according to their expected size.

### Glycosylation of antigens produced in *A. thaliana* seed

All antigens were translocated to the ER by an ER-targeting signal derived from the 2S albumin seed storage protein of *A. thaliana* [43] and retained there via a C-terminal KDEL sequence [44]. If the antigens are indeed retained in the ER, they should carry N-glycans of the high-mannose type [45]. To verify their glycosylation status, all antigens (18 in total) were deglycosylated by treatment with either endoglycosidase H (endoH) or peptide-N-glycosidase F (PNGaseF), which differ in their substrate specificity. EndoH acts on high-mannose N-glycans only [46], whereas PNGaseF processes all N-glycans except core α1,3-fucosylated structures. In plants, this modification occurs in the late Golgi apparatus and can be found on hybrid and complex N-glycans [47]. When the antigen carries ER-typical high-mannose N-glycans, it will be deglycosylated by both enzymes. However, if the antigen is present in other compartments of the secretory pathway and its glycans undergo maturation into hybrid or complex type glycans, then treatment with endoH and PNGaseF will yield differential deglycosylation, except for α1,3-fucosylated glycans that will not be processed by either of both glycosidases. However, glycoproteins carrying these structures will be resilient to digestion and hence, can be monitored as well.

**Figure 3:**
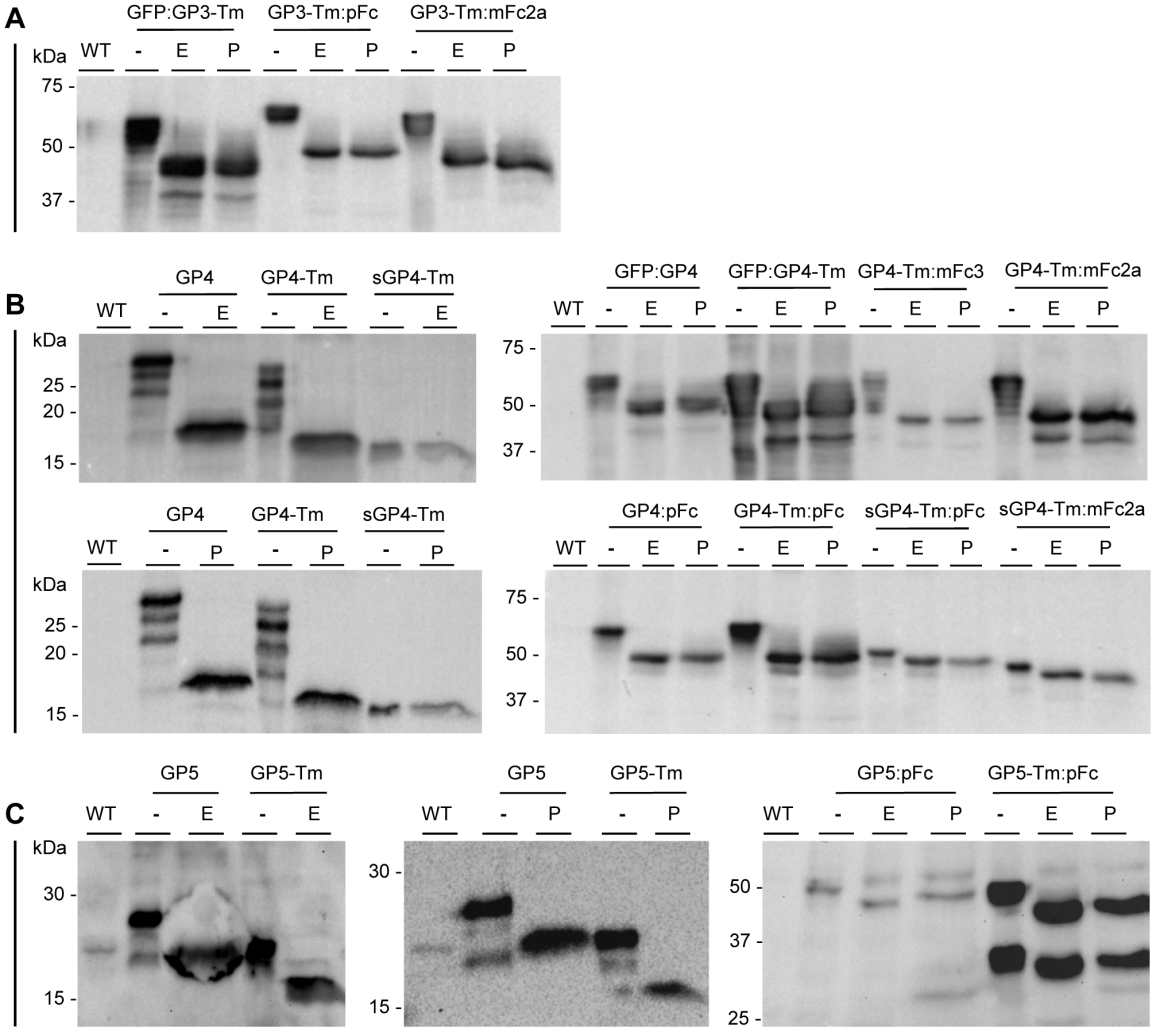
Glycan analysis of the GP3, GP4 and GP5 antigens. Seed extracts from homozygous lines (unless stated differently) producing the different antigen formats were treated with endoH or PNGaseF and analyzed by western blotting under reducing conditions. Thirty µg of TSP was loaded. (A) Glycan analysis of the GP3 antigens detected with the α-GP3 mAb VII2D [41]. (B) Glycan analysis of the GP4 antigens detected with the α-GP4 mAb XVI 11c [42]. For the sGP4-Tm, sGP4-Tm:pFc and GFP:GP4, a segregating T2 stock was used. (C) Glycan analysis of the GP5 antigens detected with an antibody against the His_6_-tag. For the GP5-Tm:pFc, a segregating T2 stock was analyzed. Abbreviations used: -, no treatment; E, endoH treatment; P, PNGaseF treatment: WT, non-treated wild-type seed extract; kDa, kilodalton.

All antigens are susceptible to deglycosylation by endoH and PNGaseF and no differences can be discerned between treatment with either glycosidase, indicating that the proteins carry high-mannose N-glycans (Figure 3A, B and C). As hybrid and/or complex glycans have been reported on KDEL-tagged recombinant proteins before [25], [48], it is possible that minute quantities of these are present on the antigens as well and that the mobility shift assay did not reveal these. However, the obtained results clearly identify high-mannose N-glycans as the predominant glycoforms. For GFP:GP3-Tm some degradation was observed (Figure 3A). Figure 3B shows that the banded pattern of both GP4 and GP4-Tm was reduced to a single band corresponding to the fully deglycosylated antigen, confirming that it indeed represents different glycoforms. Figure 3B also shows some non-reduced multimers and degradation for GFP:GP4-Tm and GP4-Tm:mFc2a. Similarly, for the GP5 antigens no differential behavior could be observed when treated with endoH or PNGaseF (Figure 3C). Deglycosylation of GP5 and GP5-Tm showed that the faint bands present before treatment correspond to partially and unglycosylated antigens, since both deglycosylation treatments resulted in the reduction of this pattern to one single band (Figure 3C). Given that the pattern of GP5-Tm:pFc (right panel, Figure 2C and 3C) was not reduced to one single band after endoH or PNGaseF treatment, the lower band does not correspond to the unglycosylated protein, but to a cleavage product (Figure 3C right panel).

To conclude, the antigens were post-translationally modified by N-glycosylation and glycosidase treatment showed that all antigens are decorated with ER-typical high-mannose N-glycans, indicating their correct production in the ER.

### Accumulation levels of antigens produced in *A. thaliana* seed

ELISAs were performed to determine the accumulation level of the antigens in the transgenic seed and to assess the effect of fusing them to stabilizing protein domains (Table S1). The antigen content was expressed as antigen mass per gram of dry seed and, after measuring the protein concentration of the seed extracts, as percentage of TSP. For most antigens, T3 homozygous (Ho) lines were analyzed. When not available, the T2 segregating (Se) seed stock was used (Table S1). Therefore, it should be taken into account that, due to a gene dosage effect [22], it is possible that the accumulation of the antigens, for which a segregating seed stock was analyzed, could increase when these lines are propagated to homozygosity. During the ELISA experiments, no signal could be obtained for GP5 and GP5:pFc. However, western blotting clearly showed that they were present in seed extracts (Figure 2C, right panel), and thus GP5 and GP5:pFc antigen accumulation was determined by signal quantification after western blot analysis via the His_6_-tag (data not shown).

**Figure 4:**
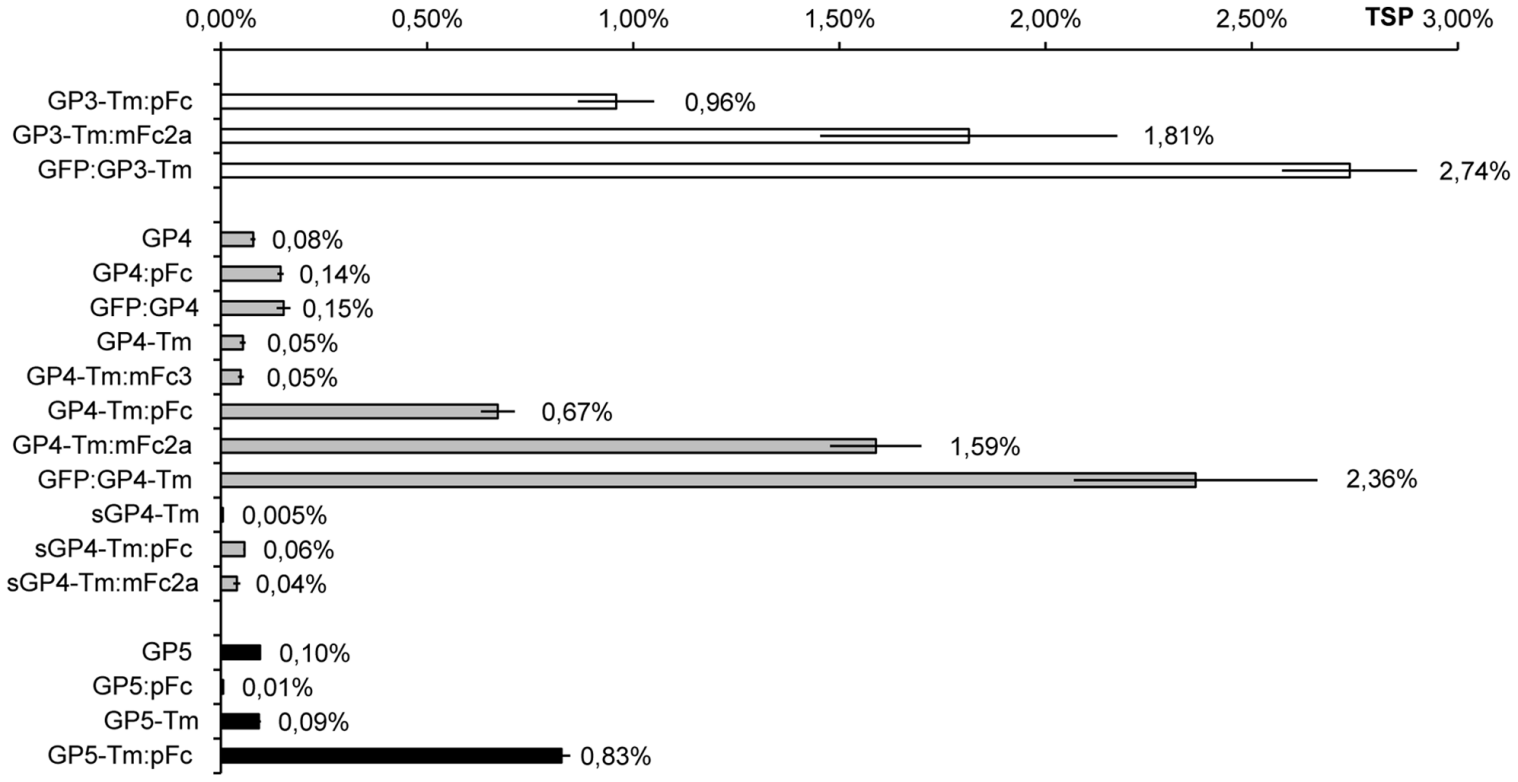
Antigen accumulation in transgenic seeds as determined by ELISA and given as percentage of TSP. Values are the mean of three biological repeats, error bars represent the standard deviation. Accumulation of the GP3, GP4 and GP5 antigens is depicted as white, grey and black bars, respectively. Abbreviation used: TSP, total soluble protein.

A graphical representation of the accumulation of the antigens (given as percentage of TSP, Figure 4) shows that there was a large variation, ranging from 0.005% of TSP for sGP4-Tm to 2.74% of TSP for GFP:GP3-Tm. Some trends could however be observed. The full-length antigens GP4 and GP5 did not accumulate well (0.08% and 0.10% of TSP, respectively) and removing their Tm to generate GP4-Tm and GP5-Tm did not alter this considerably, but fusion to stabilizing protein domains increased accumulation by several factors, up to 2.36% of TSP for GFP:GP4-Tm. Fusions between GP3-Tm and the same stabilizing domains showed a similar increase, resulting in percentages as those measured for the corresponding GP4-Tm fusions. It is noteworthy that addition of an IgG Fc to the antigens without Tm can have a beneficial effect on their accumulation, as described by De Buck et al. [49], but that this is highly dependent of the Fc added. Whereas the mFc3 did not enhance GP4-Tm accumulation, the pFc, and the mFc2a even more so, notably did so, boosting accumulation from 0.05% to 0.67% and 1.59% of TSP, respectively. The full-length antigens appeared to be less prone to manipulation by addition of stabilizing domains. In contrast to GP4, where addition of the pFc and GFP had little impact on accumulation (from 0.08% to 0.14% and 0.15% of TSP, respectively), coupling of the pFc to GP5 actually reduced accumulation of the resulting GP5:pFc (from 0.10% to 0.01% of TSP). Removing the four N-glycan attachment sites of GP4-Tm in sGP4-Tm was also deleterious, since accumulation was diminished from 0.05% to 0.005% of TSP. Again, addition of the pFc and mFc2a to sGP4-Tm slightly increased accumulation. Far outperforming any other format, the N-terminal GFP fusions, i.e. GFP:GP3-Tm and GFP:GP4-Tm, accumulated to 2.74% and 2.36% of TSP, respectively.

### Purification of antigens produced in *A. thaliana* seed

Several techniques can be used for purification. Antigens fused to an IgG Fc are suitable for protein-A affinity chromatography, whereas the formats carrying a His_6_-tag can be purified by immobilized metal affinity chromatography (IMAC).

**Figure 5:**
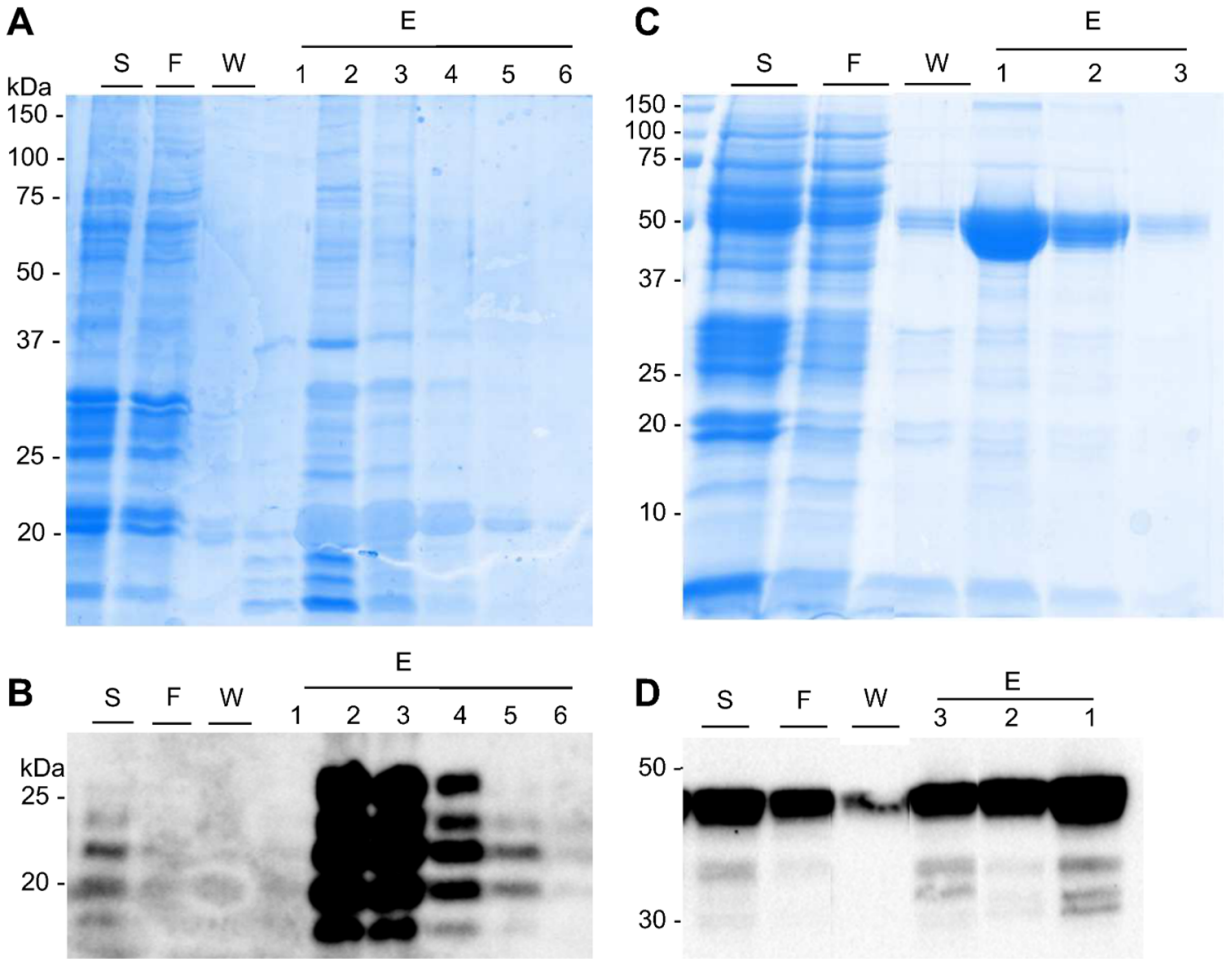
IMAC purification of selected antigens. One gram of homozygous seed stocks containing 0.14-Tm or 7.37 mg of GFP:GP4-Tm was extracted and subjected to IMAC. Purification of the GP4-Tm was analyzed by 12% SDS-PAGE and Coomassie Blue staining (A) or western blotting (B). Purification of the GFP:GP4-Tm was analyzed by SDS-PAGE (15%) and Coomassie Blue staining (C) or western blotting (D). All gels were run under reducing conditions and an antibody against the His_6_-tag was used to detect the antigens during western blot analysis. Abbreviations used: S, sample; F, flow through; W, wash; E, elution; kDa, kilodalton.

A high and low accumulating antigen, respectively GFP:GP4-Tm and GP4-Tm, were selected to be purified by IMAC. The IMAC purification of GP4-Tm resulted in fractions enriched with GP4-Tm as observed by western blot analysis (the banded pattern corresponds to the different glycoforms of GP4-Tm, Figure 5B), but GP4-Tm co-eluted with contaminating proteins as shown on the Coomassie-stained gel (Figure 5A). IMAC was thus efficient in capturing GP4-Tm, however, since the eluted fractions were only semi-pure, additional steps are necessary to obtain pure GP4-Tm. No degradation of the GP4-Tm was visible (data not shown). In contrast to GP4-Tm, the eluted GFP:GP4-Tm was of high purity (80–82%, Figure 5C). Western blot analysis (Figure 5D) showed that some GFP:GP4-Tm was still present in the flow through and after washing to remove imidazole, the final yield was only 40%. Nonetheless, successive rounds of loading the flow through onto the column and subsequent elution could increase the recovery up to 75% (data not shown). Also, small quantities of degraded GFP:GP4-Tm were visible both in the crude extract as in the purified samples (Figure 5D). These experiments suggest that when IMAC is used to purify proteins from crude *A. thaliana* seed extracts, some endogenous proteins co-purify and that the level of contaminating protein depends on the amount of target protein present in the extract. When the target protein is abundant, then it is preferentially retained by the column. Low abundant proteins on the other hand are co-purified with higher levels of endogenous proteins. In summary, IMAC is suitable to capture GP4-Tm, but additional steps are necessary to obtain pure protein. Considering GFP:GP4-Tm and taking a yield penalty into account, IMAC can be used to directly purify the antigen.

**Figure 6:**
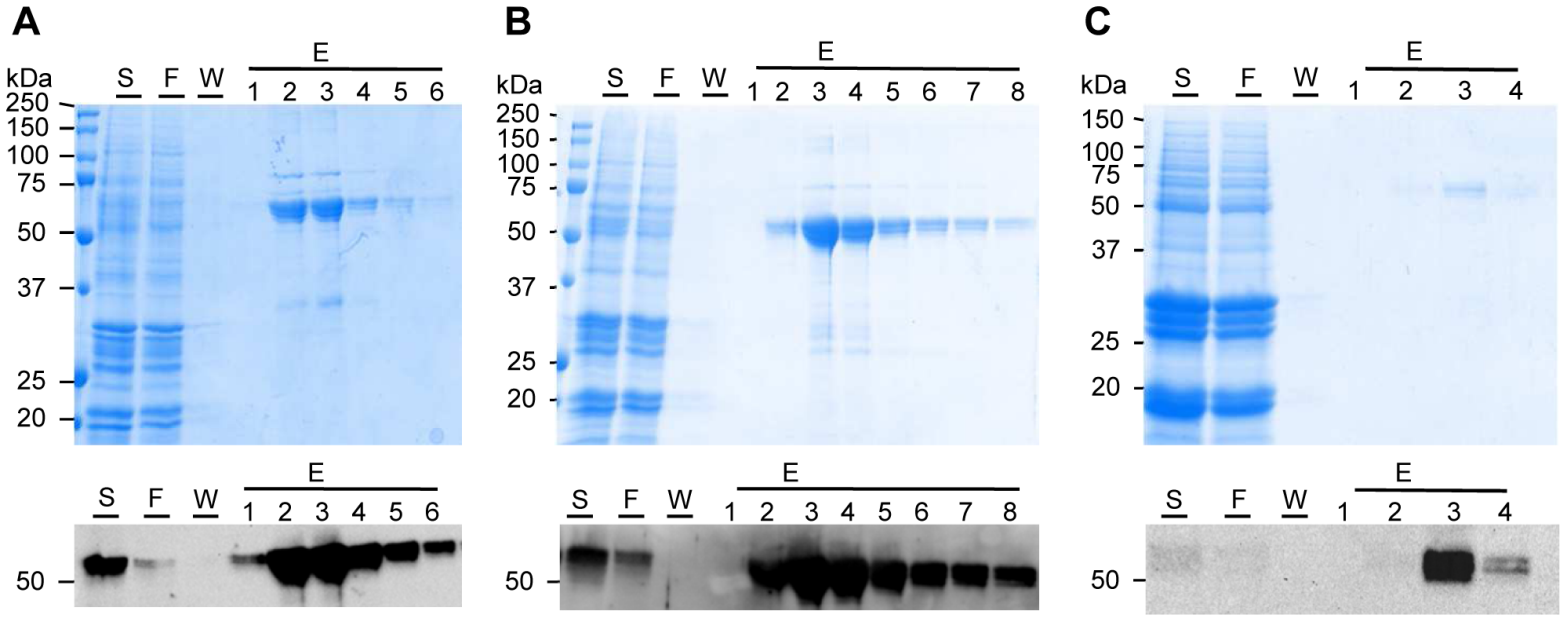
Protein-A affinity chromatography of selected antigens. For each antigen, one gram of homozygous seeds (equivalent to 0.15 mg GP4-Tm:mFc3, 2.06 mg GP4-Tm:pFc and 5.15 mg GP4-Tm:mFc2a) was extracted and subjected to protein-A chromatography. Purification of GP4-Tm:pFc (A), GP4-Tm:mFc2a (B) and GP4-Tm:mFc3 (C) was analyzed by 12% SDS-PAGE and Coomassie Blue staining (upper panels) or western blotting (lower panels). All gels were run under reducing conditions and the α-GP4 mAb XVI 11C was used to detect the antigens during western blotting. Abbreviations used: S, sample; F, flow through; W, wash; E, elution; kDa, kilodalton.

The GP4-Tm antigens fused to pFc, mFc3 and mFc2a were selected to be purified by protein-A affinity chromatography (Figure 6). The strong and specific interaction between protein-A derived from *Staphylococcus aureus* and the Fc part of the antigens should allow for efficient purification [50]. Coomassie and western blot analysis showed that all three Fcs used had affinity for the protein-A resin and that both low (GP4-Tm:mFc3 in Figure 6C) and high accumulating antigens (GP4-Tm:pFc and GP4-Tm:mFc2a, Figure 6A and B, respectively) could be captured and purified by single-step protein-A chromatography. The recovery and purity for GP4-Tm:pFc were 55 and 85%, respectively, and for GP4-Tm:mFc2a 60 and 88%, respectively (they were not determined for GP4-Tm:mFc3).

### Immunogenicity of antigens produced in *A. thaliana* seeds

**Figure 7:**
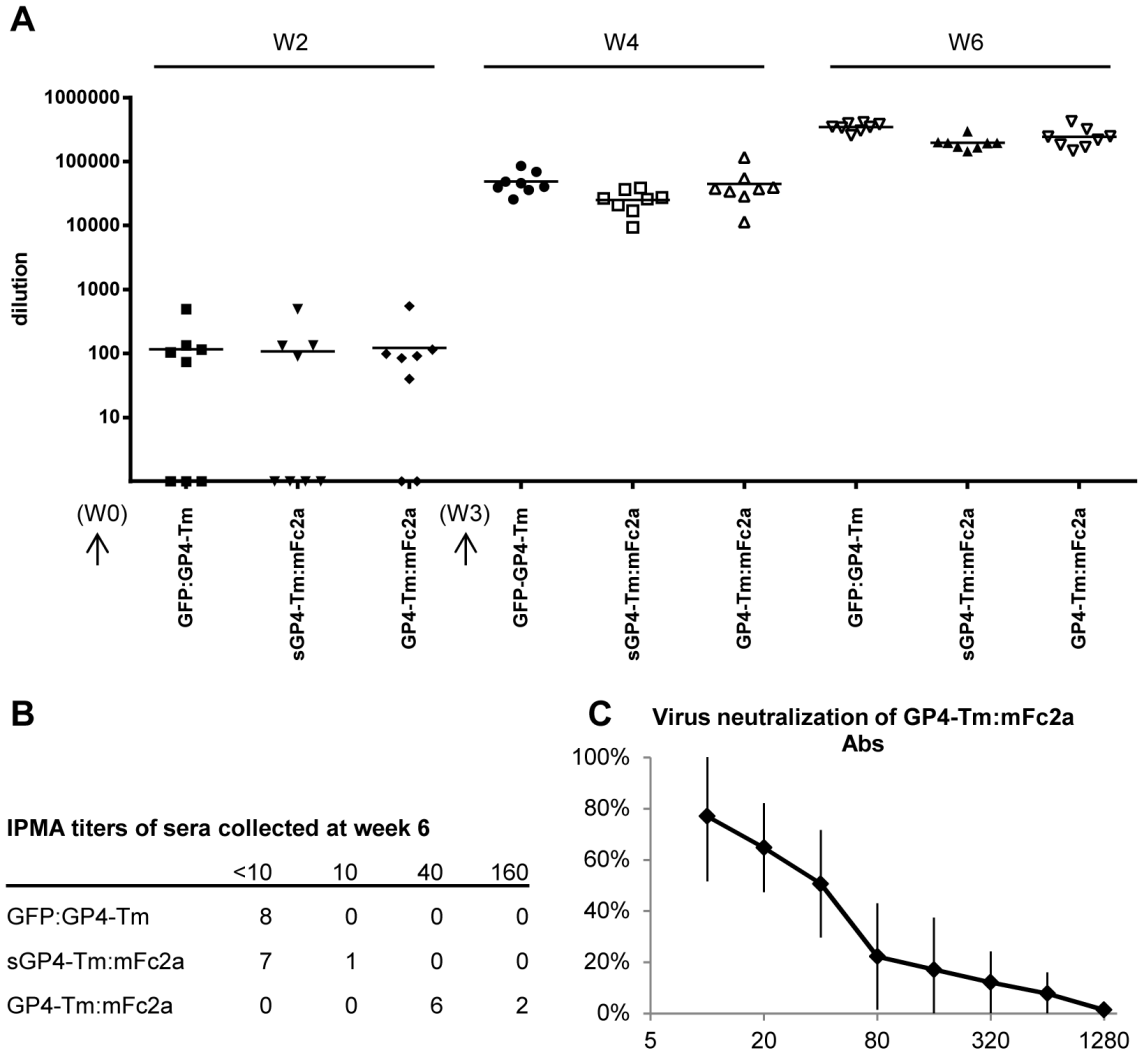
Characterization of the immunogenicity of selected GP4 antigens in a mouse model. Per antigen, eight mice were vaccinated twice with 5 µg antigen mixed with an oil-in-water adjuvant. The booster vaccination was administered at week three (both vaccinations are depicted as black arrows). (A) Antigen-specific IgG titers induced by the GFP:GP4-Tm, GP4-Tm:mFc2a and sGP4-Tm:mFc2a at weeks two, four, and six after vaccination as measured by ELISA. Per vaccinated group, the individual values for each mouse and the mean (horizontal line) are given. (B) The titers of the sera collected at week six when tested via IPMA. Per vaccinated group the number of mice with a given titer is indicated. (C) The sera at week six of the GP4-Tm:mFc2a vaccinated mice were subjected to a seroneutralization assay to determine their virus neutralizing activity. The mean percentage of virus reduction (*n* =  eight mice) in function of the dilution is given, error bars represent the standard deviation.

To study the immunogenicity of the plant-produced antigens, GFP:GP4-Tm, GP4-Tm:mFc2a and sGP4-Tm:mFc2a, all carrying the GP4-Tm, were selected to vaccinate mice. The GP4-Tm was chosen as a model antigen as it contains a B-cell epitope that consistently induces neutralizing antibodies upon infection, and neutralizing antibodies are considered a good correlate for protection against PRRSV [14], [40]. Mice were injected with 5 µg of purified antigen mixed with an oil-in-water adjuvant following a prime-boost protocol with three weeks between both vaccinations. Figure 7A shows that after six weeks high titers of GP4-Tm-specific antibodies were induced by all three antigens as measured by ELISA. The sera collected at week six were then subjected to an immuno peroxidase cell monolayer assay (IPMA) to check for viral antigen recognition (Figure 7B). Despite the high antibody titers induced by all antigens, only GP4-Tm:mFc2a generated antibodies capable of PRRSV recognition in IPMA. Subsequently these sera were shown to have virus neutralizing capacity in a seroneutralization test (Figure 7C). All three antigens thus raised high levels of antigen-specific antibodies in a mouse model, and those of the GP4-Tm:mFc2a vaccinated group were also able to neutralize the PRRSV.

**Figure 8:**
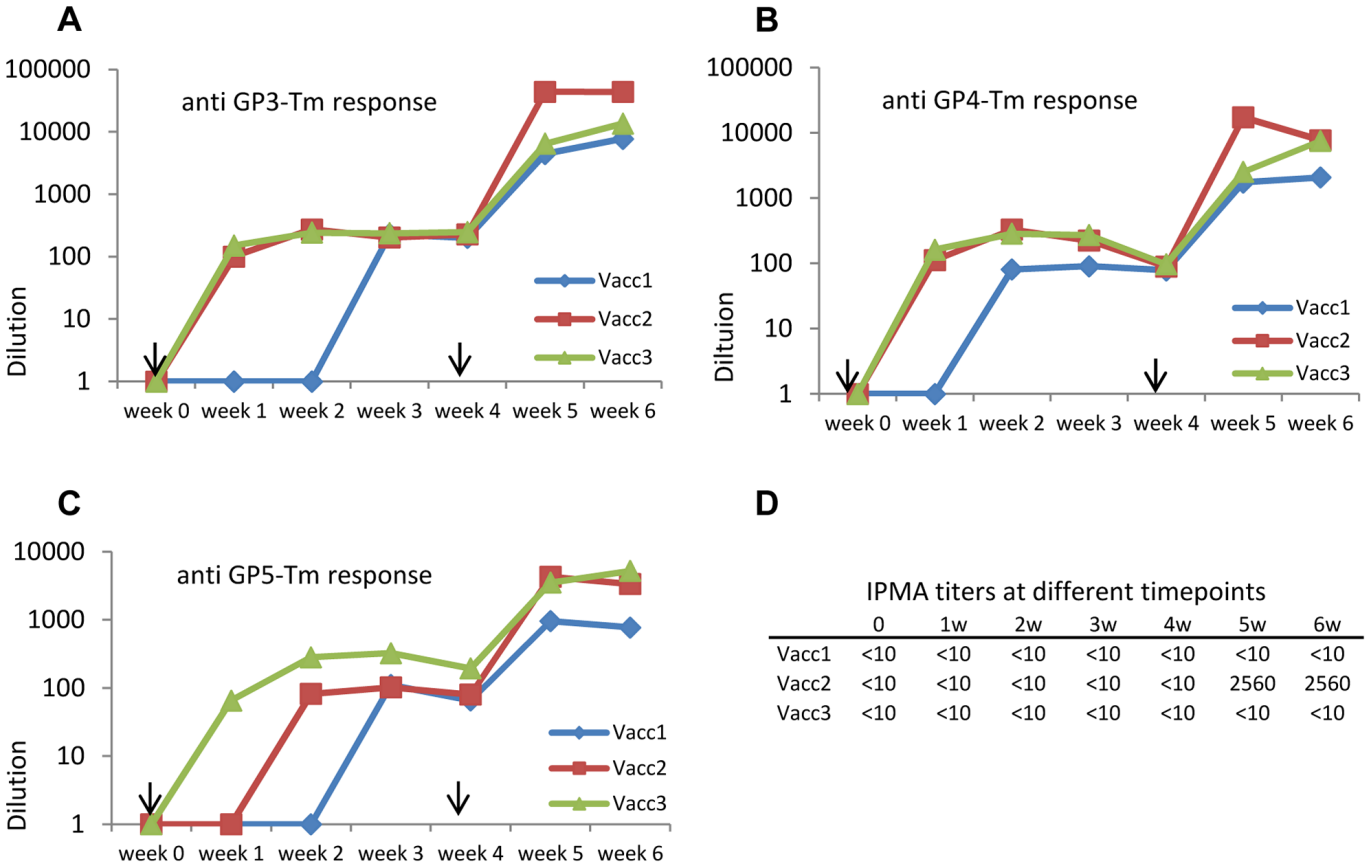
Characterization of the immunogenicity of selected antigens carrying the pFc in a porcine model. Three piglets were vaccinated twice with an antigen cocktail consisting of purified GP3-Tm:pFc, GP4-Tm:pFc and GP5-Tm:pFc mixed with an oil-in-water adjuvant. The booster vaccination was administered at week four (both vaccinations are depicted as black arrows). At different time points, the titers of IgG antibodies specific for GP3-Tm (A), GP4-Tm (B) and GP5-Tm (C) was determined. The collected sera were also tested via IPMA and titers of sera collected from week zero to six given (D).

Upon completion of the murine vaccination trial, a second study was initiated to assess the immunogenicity of antigens carrying the pFc in pigs, the *in vivo* host of the PRRSV. During this preliminary trial three piglets were vaccinated with an antigen cocktail consisting of 100 µg purified GP3-Tm:pFc, 100 µg purified GP4-Tm:pFc and 100 µg purified GP5-Tm:pFc mixed with an oil-in-water adjuvant following a prime-boost protocol with a four-week interval between both vaccinations. High GP3-Tm, GP4-Tm and GP5-Tm-specific antibody titers were obtained after the booster vaccination from week four onward (measured by ELISA; Figure 8A, B and C). When sera were tested via IPMA, high titers were found for one piglet from week five onward, however, no response could be detected for the other two piglets (Figure 8D). Sera from all three piglets collected at week five and six were subjected to a seroneutralization test, but no response above the threshold could be detected (data not shown).

In summary, all antigens vaccinated in mice and piglets generated high titers of antigen-specific antibodies after the booster vaccination and the sera of mice vaccinated with GP4-Tm:mFc2a also had virus neutralizing capacity. In contrast to this, no such activity could be detected in any of the sera of the vaccinated piglets.

## Discussion

We designed, cloned and successfully expressed a set of antigenic proteins derived from the GPs of the PRRSV EU-prototype LV in the seed of *A. thaliana*. Analysis of antigen glycosylation status showed that the antigens were post-translationally modified by N-glycosylation, and that they carried high-mannose N-glycans, corresponding to their production in the ER and possibly, also indicating their retention there. For GP4 this scenario would mean that its single-span transmembrane domain [10], predicted to leave the KDEL-tag in the cytosol out of reach of the KDEL receptor, is not correctly inserted into the ER membrane or, that GP4 is retained in the ER in a KDEL-independent manner. In animal cells, GP4 is withheld in the ER until it associates with GP2 and GP3 to form a heterotrimer after which the complex is released [39] and a similar mechanism might function in the seed. For GP5, the exact topology of the Tm, which is predicted to span the ER membrane anywhere from one to three times [10], is not known, but the presence of high-mannose glycans would strengthen the hypothesis that the KDEL-tag is functional.

On the other hand, we cannot exclude that the antigens have migrated to other subcellular compartments, since recombinant proteins with ER-typical glycosylation have been found in non-ER compartments before in the seed of Arabidopsis. Using the same expression vector, the anti-HIV monoclonal antibody 2G12 was found almost exclusively in protein storage vacuoles [25], whereas a synthetic antibody based on the 2G12 was deposited in newly formed ER-derived vesicles [26] and another synthetic antibody even in the periplasmic space [23]. All these recombinant proteins carried the KDEL sequence and were decorated to some extent with high-mannose glycans, showing that the mere presence of these glycans is not sufficient to allocate a protein to the ER. Further localization studies are thus needed to pinpoint the exact subcellular compartment in which the antigens reside.

Despite the advantages offered by the production of recombinant proteins in plants, such as safety, flexible scalability and complex protein synthesis, low protein yields have been a major problem during previous studies concerning PRRSV antigens [31]–[36]. Antigen accumulation ranged from 0.011% of TSP for recombinant GP5 expressed transiently in tobacco leaves [34] to 0.65% of TSP for the N protein produced in soybean seed [36]. To overcome this hurdle, we included in our set of antigenic proteins a number of antigen formats carrying stable protein domains, such as GFP or an immunoglobulin Fc fragment, which are expected to increase antigen accumulation. Indeed, a 6-fold increase (taking into account the similar molecular weight of GP4-Tm and the pFc domain) in accumulation of the GP4-Tm component was observed when comparing the GP4-Tm antigen (0.05% of TSP) to the GP4-Tm:pFc (0.67% of TSP), rising to over 15-fold for the GP4-Tm:mFc2a (1.59% of TSP) and even more than 20-fold when comparing to the GFP:GP4-Tm (2.36% of TSP). Considering the GP5 antigens, both GP5 and GP5-Tm accumulated to about 0.09% of TSP and this rose to 0.83% of TSP for GP5-Tm:pFc, corresponding to 2.3 mg/g of seed weight. These numbers are considerably higher than those obtained for recombinant GP5 produced in banana (0.037% of leaf TSP [31]), potato (4.7 µg/g of leaf fresh weight [32]), tobacco (0.011% of TSP [34]) or for a fusion between GP5 and the B subunit of *Escherichia coli* heat-labile enterotoxin produced in tobacco (0.015% of TSP [33]).

Further, our results are in line with those obtained by Obregon et al. [51] who fused the HIV-1 p24 antigenic protein to the Fc of human IgA, resulting in a 13-fold increase in antigen accumulation and those of De Buck et al. [49] who could boost the accumulation of Nanobodies® (antigen-binding domains of camelid heavy-chain only antibodies) in the seed of *Arabidopsis* by 10- to 100-fold up to 16% of TSP after fusion with Fcs of different origin. As a general tendency we observed that adding stabilizing domains to the truncated antigens GP3-Tm, GP4-Tm and GP5-Tm had a positive influence on antigen accumulation and that the extent of this influence depended on the domain added, with GFP giving the highest accumulation followed by, in decreasing order, the mFc2a, pFc and mFc3.

GFP has a long history in the field of cell biology and GFP-fusion proteins have been made for decades [52]. The main goal of these fusions has always been to create reporter proteins that could be followed at the cellular level. However, our data indicate that GFP can also effectively be used to augment target protein accumulation and, taken into account its stability and the fact that several GFP-based purification methods have been published [53], [54], fusion to GFP could represent an alternative strategy to boost target protein production. However, caution should be taken in the context of vaccination where GFP might interfere with the immune response raised against the GFP-tagged antigen.

Manipulation of the full-length antigens GP4 and GP5 by addition of stable protein domains was much less effective, indicating that the Tm somehow compromises antigen accumulation. Mutating the N-glycan attachment sites of the GP4-Tm profoundly disturbed accumulation of the resulting deglycosylated sGP4-Tm. Both sGP4-Tm, as well all fusions herewith, underperformed when compared to the corresponding glycosylated formats. It will be interesting to determine whether this is due to the change in primary structure after amino acid substitution or because of the absence of N-glycans, possibly resulting in a perturbation of interaction with chaperones and the quality control mechanism in the ER. In summary, we observed that stable protein domains can be added to the antigens as a strategy to increase antigen accumulation with GFP being most beneficial, that the effect of such fusions depends on both the antigen and the stable domain, and that for each antigen the optimal manipulation should therefore be identified on a case by case basis.

In addition to boosting accumulation, the Fc domains also allow for protein-A affinity chromatography. We showed that antigens carrying the pFc, mFc3 and mFc2a and varying in accumulation between 0.15 and 5.15 mg per gram dry seed could all be purified by single-step protein-A affinity chromatography with a recovery of about 60%. On the other hand, single-step IMAC purification of antigen formats with a His_6_-tag was restricted to high abundant antigens such as GFP:GP4-Tm. It seems that some seed proteins also show affinity for the resin and compete for binding sites on the IMAC column. If the antigen is low abundant, then more endogenous proteins will bind and co-elute, lowering the final antigen purity and increasing the need for an additional purification step.

The platform to be chosen for subunit vaccine production should allow for high production and correct folding and assembly of the antigens. Regarding the latter, it is doubtful that complex PRRSV antigens can be produced correctly in bacteria. Indeed, disease enhancement was observed when recombinant GP5 from *E. coli* was vaccinated [55]. PRRSV antigens were also produced in baculovirus infected insect cells, but only low yields were achieved [56]. Virus-like particles containing GP5 or GP5 and M could be isolated from the insect cell culture medium, but no yield parameter was reported [57], [58]. Also when PRRSV antigens were produced *in planta,* accumulation was rather low, with a maximum of 0.65% of TSP when the N protein was produced in soybean seeds [36]. In contrast to previous studies, we managed to produce several PRRSV-derived antigens to levels of 1% of TSP and higher, a level considered to be economically relevant [38], and extracted these to high purity. Subsequently, the immunogenicity of the plant-produced antigens was assessed in a murine model. The GFP:GP4-Tm, GP4-Tm:mFc2a and sGP4-Tm:mFc2a were vaccinated in mice and although all antigens generated high titers of antigen-specific antibodies, only those induced by GP4-Tm:mFc2a were virus neutralizing. Whereas both GFP and mFc2a served to increase antigen accumulation in the seed, the latter also seemed to function as an immune enhancing tag, possibly by targeting the antigen to antigen-presenting cells via interaction with Fc receptors [59], [60]. Deletion of the N-glycans by amino acid substitution was detrimental for antigen immunogenicity, as the sGP4-Tm:mFc2a did not induce virus neutralizing antibodies. The ER-associated high-mannose glycans thus seem beneficial for antigen immunogenicity. Indeed, mannosylation of antigens has been shown to selectively target antigen presenting cells, thereby enhancing antigen immunogenicity [61], [62]. In short, GP4-derived antigen is capable of eliciting virus neutralizing antibodies in a murine model, confirming the immunogenicity of the plant-produced antigens. Upon completion of the murine trial, antigen immunogenicity was also preliminary assessed in a porcine model. Three piglets receiving an antigen cocktail consisting of purified GP3-Tm:pFc, GP4-Tm:pFc and GP5-Tm:pFc developed high titers of antigen-specific antibodies, but only the sera of one piglet recognized the PRRSV in the IPMA and no virus neutralizing activity could be detected. In this respect it is interesting to mention the findings of Plana-Durán et al. [63] who observed that pigs having received an inactivated PRRSV vaccine were protected upon challenge even though their sera were unresponsive in IPMA before challenge. It has also been shown that some experimental, inactivated PRRSV vaccines confer partial protection even without the presence of virus neutralizing antibodies prior to challenge [64]. Due to a priming effect these vaccinated piglets generate a faster and stronger neutralizing antibody response after challenge. Taken these considerations into account, it becomes clear that the antigens need to be further examined via a challenge experiment to study their capacity to prime the antibody response, stimulate a cellular response and reduce viremia. This will allow a final assessment of the protective capacity of a plant-produced PRRSV vaccine.

In conclusion, we generated a set of antigenic proteins derived from the PRRSV, incorporating elements known to be important in immunity against PRRSV, in *A. thaliana* seed. The antigens were correctly produced in the ER, accumulated to levels that are economically relevant and could be purified by single-step affinity chromatography. The immunogenicity of the plant-produced antigens was confirmed in a murine model and preliminary tested in a porcine model. It is now time to perform a challenge experiment to assess the protective capacity of the plant-produced antigens and continue on the road toward a safe and effective PRRSV vaccine.

## Materials and Methods

### Ethics statement

All animal experiments were approved by the Local Ethical Committee of the Faculty of Veterinary Medicine, Ghent University.

### Cloning and generation of transgenic lines

The original sequences of the GP3, GP4 and GP5 genes were derived from the complete LV-genome (GenBank accession number M96262.2) and all the cloned genes described in the following paragraphs were deposited at GenBank (accession numbers KF983786 to KF983804).

All sequences coding for the antigens containing GP4, GP5 and their truncated counterparts GP4-Tm, sGP4-Tm, and GP5-Tm were cloned in the seed-specific expression cassette of the gateway compatible pPphasGW destination vector [37] containing the β-*phaesolin* promoter, the *arcelin-5I* terminator and the *nptII* gene for kanamycin resistance [22]. The sequences of the antigens were further complemented at their 5′ end with the Kozak motif (CCACC) plus the coding sequence of the ER-targeting signal peptide from the *Arabidopsis* 2S seed storage protein and at their 3′ end with the coding sequence of the KDEL ER-retention signal. The genes of the GP4, GP5 and truncated formats GP3-Tm, GP4-Tm, sGP4-Tm and GP5-Tm were chemically synthesized flanked by the gateway recombination sites attB1-B2. Additionally, a sequence coding for six histidines (His_6_-tag) was inserted upstream of the KDEL coding sequence. A BP recombination reaction with the pDON221 and LR reaction with the pPhasGW allowed transfer of all genes to the destination vector, except those coding for the GP3-Tm and fusions thereof. The latter were cloned into a multisite gateway destination vector using the aforementioned regulatory elements and complemented with the same sequences encoding the ER-targeting and retention signal, resulting in an expression vector bearing the *bar* gene as resistance marker (all Gateway reactions were performed according to the Gateway manual protocol). The codon use of all cloned sequences was optimized to resemble that displayed by *Arabidopsis* seed storage proteins and all genes were sequence verified before transfer to the destination vectors.

Coupling of stabilizing domains to the GP4, GP5 and truncated formats GP3-Tm, GP4-Tm, sGP4-Tm and GP5-Tm was done via classical cloning of the corresponding coding sequences. The sequence of the porcine IgG3 Fc (accession no: EU372658; pFc) was added to that of the antigens using a Gateway compatible pDON221 vector that contained the gene of a camel-like antibody (a fusion between the antigen-binding domain of a heavy-chain camel antibody with an Fc fragment of choice) carrying the porcine IgG3 Fc [65]. The sequence of the antigen binding domain was swapped by restriction digest with the enzymes *EcoR*I and *Avr*II and replaced by that of the desired antigen. To this goal the sequences of the GP4, GP5, and truncated counterparts, were amplified by polymerase chain reaction (PCR) to include the *EcoR*I and *Avr*II restriction sites (Table S2 and S3). An LR reaction transferred the resulting genes to the pPhasGW.

A similar strategy was followed to incorporate the sequences of the murine IgG3 Fc (NCBI accession no X00915; UniProt accession no P03987; mFc3) and IgG2a Fc (derived from the full-length murine IgG2a heavy chain; NCBI accession no AAB59660.1). The sequences of the antigen binding domains of camel-like antibodies with murine IgG3 Fc and IgG2a Fc were swapped by restriction digest with *EcoR*I and *Sac*II and substituted by those of selected antigens that had been amplified by PCR (Table S2 and S3). In contrast however to the cloning of the porcine IgG3 Fc, the sequence coding for the His_6_-tag was no longer included in the final product. Subsequently, genes were transferred to the destination vectors by LR reaction.

Concerning the GFP fusions, the sequence of the enhanced green fluorescent protein (derived from the pFS7(GW) [66]) was extended *in silico* at its 5′ end with the Kozak and 2S sequence. At its 3′ end the coding sequences of the his_6_-tag, the GP4-Tm plus KDEL and a *BamH*I restriction site were added. The resulting GFP:GP4-Tm gene was embedded between the attL1–2 sites and chemically synthesized. By PCR it was extended to form the GFP:GP4 gene (Table S2 and S3). The GP4-Tm sequence in the GFP:GP4-Tm gene was swapped by restriction digest with *Sac*II and *BamH*I for an amplicon of the GP3-Tm generating the GFP:GP3-Tm gene (Tabel S1 and S2). LR reaction transferred all genes to the destination vectors.

Heat shock delivered all expression vectors to *Agrobacterium* strain C58C1Rif^R^ containing the pMP90 virulence plasmid [67] and the resulting strains were used to transform *Arabidopsis thaliana* (L.) Heyhn, ecotype Columbia 0, by floral dip [68]. Transformed seeds containing the T-DNA were identified and lines propagated to homozygosity as detailed in [68]. In short, T1 seeds obtained after floral dip were grown on selective Murashige and Skoog medium for three to four weeks at 21°C on a 16-h light/8 h dark cycle. Twenty transformants were then transferred to soil and grown under the same conditions. These were selfed and when seed setting was complete, transferred to a room at 25°C and low humidity until completely dry. The dry seeds of each transformant were harvested to obtain twenty T2 seed stocks. These stocks were screened for the presence of antigen by western blot analysis as detailed below and seven stocks with high antigen accumulation were analyzed for the number of integrated T-DNA loci. As the T1 transformants are per definition hemizygous, the T-DNA locus number can be determined by the segregation ratio in the T2 generation. To identify the single locus T-DNA integration lines, seeds of segregating T2 stocks were sown on selective medium and the observed ratio of germinated over sensitive seeds was compared to the expected 3:1 segregation ratio for single locus lines using a χ^2^-statistical test [68]. For each antigen the T2 stock with the highest antigen content and the T-DNA integrated at a single locus in the genome was retained and propagated to homozygosity.

### Protein extraction, protein concentration determination, SDS-PAGE, Coomassie staining and western blot analysis

To characterize the antigen production in transgenic seed, TSP was extracted as described elsewhere [68] with minor modifications: 1 ml of cold extraction buffer (50 mM Phosphate buffer, pH 7.8, 0.3 M NaCl, 0.1% (w/v) 3-[(3-Cholamidopropyl)dimethylammonio]-1-propanesulfonate hydrate (CHAPS) supplemented with an ethylenediaminetetraacetic acid (EDTA) free, proteinase inhibitor cocktail (1 tablet per 50 ml; Roche)) was added to 10 mg of liquid nitrogen (N_2_) frozen, crushed seeds. Samples were vortexed for 1 minute and centrifuged at approximately 20,000 g and 4°C for 30 minutes. 700 µl supernatant was removed, snap-frozen in liquid N_2_ and stored at −20°C. Total protein concentration was determined by the Lowry method as described in [68].

Seed extracts containing TSP were separated by reducing sodium dodecyl sulfate-polyacrylamide gel electrophoresis (SDS-PAGE). To visualize proteins by Coomassie staining, the gels were submerged in InstantBlue^TM^ (Expedeon) solution for an hour and then washed three times for 20 minutes with double distilled water (ddH_2_O). Stained gels were photographed with the Molecular Imager® ChemiDoc^TM^ XRS+ Imaging System (Biorad). For western blot analysis the proteins were transferred electrophoretically from a polyacrylamide gel onto Immobilon-P polyvinylidene fluoride membrane (Millipore, Billerica, MA). Blots were blocked in 4% (w/v) skimmed milk and 0.1% (v/v) Tween 20 in phosphate-buffered saline (PBS). Several commercial secondary antibodies coupled to horse radish peroxidase (HRP) were used to develop the western blots. Anti-mouse IgG HRP-linked whole antibody (from sheep, GE healthcare) was used in combination with the α-GP4 mAb XVI11C and α-GP5 mAbs II5D, II5A and VII2H at a dilution of 1∶5000 in blocking buffer. The α-GP4 mAb XVIII5G and α-GP3 mAb VII2D were detected with α-mouse IgG1 (gamma-chain)-HRP (from rabbit, Sigma Aldrich) diluted 1∶5000 in blocking buffer. As an antibody against the His_6_-tag, a penta-His HRP conjugate (Qiagen) was used diluted 1∶2000 in blocking buffer. Western blotting was further performed as described in [68] and the released chemiluminescent signal measured with the abovementioned imaging system. Quantification of signal intensities was performed with the Image Lab 3.0 software (Biorad).

### Glycan analysis

Seed extracts containing TSP were treated with peptide N-glycosidase F (PNGaseF, New England Biolabs) and recombinant endoglycosidase H (endoH, New England Biolabs) to analyze antigen glycosylation status. Glycosidase reactions were performed according to the manufacturer's protocol. In short, to 9 µl of extract, 1 µl of 10× denaturing buffer was added and the sample was incubated at 100°C for 10 minutes. Then, for the PNGaseF digest, 2 µl G7-buffer, 2 µl 10% NP-40 and 2 µl PNGaseF was added to the sample whereas for the endoH digest, 2 µl reaction buffer and 1 µl endoH was added. Sample volume was adjusted to 20 µl with ddH_2_O, after which the sample was incubated at 37°C for one hour and analyzed by western blotting.

### Antigen purification by IMAC

The GP4-Tm and GFP:GP4-Tm antigens were purified by IMAC on an Äkta Explorer® purification system fitted with a His Trap® Fast Flow column (1ml, GE healthcare). Seed extracts were prepared in binding buffer (50 mM phosphate buffer pH 7.4, 0.3 M NaCl, 20 mM imidazole, 0.1% CHAPS supplemented with an EDTA-free proteinase inhibitor cocktail (1 tablet per 50 ml; Roche)) by adding frozen and pulverized seeds to cold buffer at a fixed w/v of 1 g seeds per 70 ml buffer. The extract was vortexed and centrifuged for 30 minutes at 4°C and 40,000 g to remove cell debris. The supernatant was removed, passed through a GF-prefilter (Sartorius) and a Millex®-HP filter unit (Millipore) and then flowed over the IMAC column. After washing the column with binding buffer, antigens were eluted with 0.5 M imidazole (in binding buffer). To remove the imidazole, the antigens were washed with PBS on Amicon® Ultra centrifugal filters (Millipore, 3 kDa molecular weight cutoff). Antigen purity was determined by densitometric analysis of Coomassie-stained polyacrylamide gels using the aforementioned imaging system and the Image Lab 3.0 software (Biorad). Antigen concentration was derived from the sample optical density at 280 nm measured by a NanoDrop® ND-1000 spectrophotometer.

### Antigen purification by protein-A affinity chromatography

To purify antigens carrying an Fc chain (mFc3, mFc2a or pFc) protein-A affinity chromatography was performed as described in [49] with minor modifications. Seed extracts were prepared in binding buffer (50 mM phosphate buffer pH 7.4, 0.3 M NaCl, 0.1% CHAPS supplemented with an EDTA-free proteinase inhibitor cocktail (1 tablet per 50 ml; Roche)) by adding frozen and pulverized seeds to cold buffer at a fixed w/v of 1 g seeds per 70 ml buffer. The extract was vortexed and centrifuged for 30 minutes at 4°C and 40,000 g to remove cell debris. The supernatant was removed, passed through a GF-prefilter (Sartorius) and a Millex®-HP filter unit (Millipore) and then flowed over the protein-A column (MabSelect SuRe 1 ml; GE Healthcare) fitted onto Äkta Explorer® purification system. After washing the column with binding buffer, antibodies were eluted with 1 M arginine buffer (pH 2.7) and immediately neutralized with 1 M TrisHCl (pH 9.0). To remove the arginine, the antigens were washed with PBS on Amicon® Ultra centrifugal filters (Millipore, 3 kDa molecular weight cutoff). Antibody concentration was derived from the sample optical density at 280 nm measured by a NanoDrop® ND-1000 spectrophotometer.

### Antigen purification by gel filtration

After an initial chromatography step, antigens to be used in ELISA and in the vaccination trials were further subjected to gel filtration to remove remaining impurities. To this goal, IMAC or protein-A elution fractions were passed over a Superdex-200 column (GE Healthcare) fitted onto an Äkta Explorer® purification system with lipopolysaccharide-free PBS as the mobile phase. Removal of impurities and/or degradation products was assayed by Coomassie staining of polyacrylamide gels and whenever necessary, pure, gel-filtrated antigen was concentrated using Amicon® Ultra centrifugal filters (Millipore, 3 kDa molecular weight cutoff). The antigen concentration was derived from the sample optical density at 280 nm measured by a NanoDrop® ND-1000 spectrophotometer.

### ELISA to determine antigen accumulation in the seed

The antigen content of the seed of high accumulating lines was analyzed by ELISA. Seed extracts were prepared as detailed above, dilution series were made in duplicate with PBS as coating buffer, loaded onto Maxisorp 96-well plates (Nunc, Sigma Aldrich) and coated overnight at 4°C. Wells were washed three times with 270 µl washing buffer (PBS, 0.1% (v/v) Tween-20) and this was repeated between all subsequent steps. Remaining protein-free binding sites were blocked with 270 µl blocking buffer (PBS, 1% (w/v) bovine serum albumin (BSA, Sigma Aldrich), 0.1% (v/v) Tween-20) for 60 minutes at room temperature. 100 µl primary antibody solution was added and plates incubated for 60 minutes at room temperature. Secondary antibody solution was added (100 µl) and plates again incubated for 60 minutes at room temperature. Plates were then washed five times with washing buffer and 100 µl 3, 3′, 5, 5′-Tetramethylbenzidine Liquid Substrate (TMB, Sigma Aldrich) was added. After 10 minutes, color development was terminated by addition of 100 µl 1 M HCl and plates were read at 450 nm in a VERSAmax tunable microplate reader. Standard curves were derived from duplicate two-fold serial dilutions of purified antigen, measured values were plotted against standard curves and antigen content of the seed calculated after correcting for antigen molecular weight. For analysis of the GP3-Tm fusion antigens, the mAb α-GP3 VII2D was used (diluted 25∶1000 in blocking buffer) as primary antibody together with α-mouse IgG1 (gamma-chain)-HRP (from rabbit, Sigma Aldrich), diluted 1∶5000 in blocking buffer, as the secondary antibody and protein-A purified GP3-Tm:pFc as standard. The GP4, GP4-Tm and sGP4-Tm containing antigens were analyzed with the α-GP4 mAb XVIII5G (diluted 25∶1000 in blocking buffer) as primary antibody, the α-mouse IgG1 (gamma-chain)-HRP (from rabbit, Sigma Aldrich), diluted 1∶5000 in blocking buffer, as the secondary antibody and protein-A purified GP4-Tm:pFc as standard. For all antigens carrying GP5 or GP5-Tm, a murine anti His_6_-tag (Serotec) primary antibody was used (diluted 1∶1000 in blocking buffer), together with an α-mouse IgG HRP-linked whole antibody (from sheep, GE healthcare) as secondary antibody (diluted 1∶5000) and protein-A purified GP5-Tm:pFc as standard.

### Immunization of mice

Six- to eight-week-old female C57BL/6 mice were obtained from Janvier (Belgium). Mice were housed under specific pathogen-free conditions. All animal experiments were approved by the Local Ethical Committee of Ghent University. Mice were vaccinated twice with a 3-week interval by subcutaneous injection with either 5 µg purified GFP:GP4-Tm, GP4-Tm:mFc2a or sGP4-Tm:mFc2a mixed in a 1∶1 ratio with the squalene-based oil-in-water adjuvant AddaVax (Invivogen). The antigens were isolated and purified from crude seed extracts by IMAC (GFP:GP4-Tm) or protein-A affinity chromatography (sGP4-Tm:mFc2a and GP4-Tm:mFc2a), followed by a gel filtration step as described before. During the trial, blood samples were collected at week zero (preimmune), two, four and six post immunization. Serum samples for IPMA and detection of virus neutralizing antibodies were incubated 30 minutes at 56°C prior to freezing. Mice were euthanized after the final bleeding at week six.

### Characterization of the antibody response in mice

For detection of the α-GP4 antibodies, ELISA was performed on serum samples with purified GP4-Tm:pFc as antigen. Maxisorp 96-well plates (Nunc, Sigma Aldrich) were coated overnight at 4°C with 1 µg antigen per well (in 100 µl PBS). Wells were washed three times with 270 µl washing buffer (PBS, 0.1% v/v Tween-20) and this step was repeated between all following steps unless stated differently. Wells were blocked with 270 µl blocking buffer (PBS, 0.1% Tween-20, 1% BSA) for two hours at room temperature. 100 µl of four-fold serial dilutions (diluted in blocking buffer with an initial dilution of 1∶50) of sera were added and plates incubated for 90 minutes at room temperature. Subsequently 100 µl of goat anti-mouse IgG-HRP (Southern Biotech) diluted 1∶10.000 in blocking buffer was added and plates incubated 90 minutes at room temperature. Wells were washed five times with 270 µl blocking buffer and 100 µl TMB (Sigma Aldrich) was added. After 10 minutes color development was terminated by addition of 100 µl 1 M HCl and plates were read at 450 nm in a VERSAmax tunable microplate reader.

To determine if the antibodies elicited by vaccination were able to recognize the PRRSV, the collected sera were tested via an IPMA as described elsewhere [69]. Virus neutralizing antibody titers were detected by a single-replication virus-neutralization test as described in [64] with minor modifications. Two-fold serial dilutions of sera (starting from a 1∶10 dilution) were mixed with equal volumes of porcine alveolar macrophage-grown LV resulting in a final titer of 10^5^ TCID_50_/ml. Virus-sera mixtures were incubated for one hour at 37°C and transferred to a 96-well plate (50 µl/well) with Marc-145 cells that were cultivated 48 hours prior to use. The inoculum was removed after one hour and replaced by medium, after which the cells were incubated for another 11 hours, fixed by drying and stored at −20°C. The cells were stained for PRRSV infection with mAb 13E2 [41] against the nucleocapsid protein of PRRSV and peroxidase conjugated goat α-mouse polyclonal antibodies (Dako), followed by development with 3-amino-9-ethylcarbazole. The number of infected cells in each well was counted in three fields at 200× magnification, and expressed relative (%) to the mean number of infected cells for all mock conditions within the same experiment.

### Immunization of piglets

Three piglets four weeks of age were purchased from a PRRSV-negative farm, their PRRSV-seronegative status was confirmed by IPMA [69] upon arrival and animals were housed in isolation units with HEPA-filtered air. Heat lamps were provided during the first few weeks and piglets were fed grains sprinkled with milk powder and had access to water ad libitum. The animal experiment was approved by the local ethical committee of the Faculty of Veterinary Medicine, Ghent University. The three piglets were injected twice in the neck muscles behind the ear (intramuscular) with an experimental vaccine following a prime – boost protocol. The vaccine consisted of an antigen cocktail containing 100 µg purified GP3-Tm:pFc, 100 µg purified GP4-Tm:pFc and 100 µg purified GP5-Tm:pFc (300 µg antigen in total) mixed with an oil-in-water adjuvant in a 1∶1 ratio (Suvaxyn, Fort Dodge Animal Health). The antigens were isolated and purified from crude seed extracts by protein-A affinity chromatography followed by a gel filtration step as described before. The booster vaccination was administered at week four and blood samples (5 ml taken from the jugular vein with disposable syringes) were collected at a weekly basis from week zero (preimmune) onward. Serum samples for IPMA and detection of virus neutralizing antibodies were incubated 30 minutes at 56°C prior to freezing. Piglets were euthanized at the end of the experiment by slow injection of an overdose (15 ml) of a central nervous system depressant (Natrium Pentobarbital 20%) in the jugular vein.

### Characterization of the antibody response in piglets

Different ELISA protocols were used to measure the α-GP3, α-GP4 and α-GP5 antibodies in the piglet sera. To detect the α-GP3 and α-GP4 antibodies, purified GP3-Tm:mFc2a or GP4-Tm:mFc2a was used as antigen respectively. ELISA was further performed as described above except that 100 µl of polyclonal α-porcine IgG-HRP diluted 1∶40.000 in blocking buffer was added to the wells to detect the α-GP3 or α-GP4 antibodies. To measure α-GP5 antibodies, the wells were coated with a seed extract containing the GP5-Tm. To this goal one gram of homozygous GP5-Tm seed stock was extracted with 10 ml of PBS, the extract diluted 1∶100 in PBS and 100 µl containing 0.3 µg GP5-Tm added to the wells. Plates were incubated overnight at 4°C and protocol was followed as described for the α-GP3 and α-GP4 antibodies. To determine if the antibodies elicited by vaccination were able to recognize the PRRSV, the collected sera were tested via an IPMA as described elsewhere [69]. Virus neutralizing antibody titers were detected by a single-replication virus-neutralization test as described above.

## Supporting Information

Table S1 **Antigen accumulation level.** For each antigen given values are the mean of three biological repeats. Abbreviations used: Ho, homozygous seed stock; Se, segregating seed stock; DS, dry seed; SD, standard deviation; TSP, total soluble protein. Numbers in *italic* are derived from signal quantification after western blotting via the His_6_-tag, since the GP5 and GP5:pFc antigen could not be detected by ELISA.
(DOCX)

Table S2 **(Multistep) extension PCR data.** Are given: the cloning project involved, the target of amplification, the forward and reverse primer used in each reaction, the number of consecutive extension PCRs performed for each project and the annealing temperature of the first five and last twenty cycles. The Phusion® High-Fidelity DNA Polymerase (Finnzymes) was used for all reactions according to the manufacturer's guidelines. Abbreviations used; T ann, annealing temperature.
(DOCX)

Table S3 **Primer sequences.**
(DOCX)
