# Supplementary material for: Boosting In Planta Production of Antigens Derived from the Porcine Reproductive and Respiratory Syndrome Virus (PRRSV) and Subsequent Evaluation of Their Immunogenicity
Source: PLoS One. 2014 Mar 10;9(3):e91386. doi: 10.1371/journal.pone.0091386 (PMC3948849; doi:10.1371/journal.pone.0091386)
Supplement: Table S1 — Antigen accumulation level. For each antigen given values are the mean of three biological repeats. Abbreviations used: Ho, homozygous seed stock; Se, segregating seed stock; DS, dry seed; SD, standard deviation; TSP, total soluble protein. Numbers in italic are derived from signal quantification after western blotting via the His6-tag, since the GP5 and GP5:pFc antigen could not be detected by ELISA. (DOCX) [file pone.0091386.s001.docx]

**Table S1.** For each antigen given values are the mean of three biological repeats. Abbreviations used: Ho, homozygous seed stock; Se, segregating seed stock; DS, dry seed; SD, standard deviation; TSP, total soluble protein. Numbers in *italic* are derived from signal quantification after western blotting via the His_6_-tag, since the GP5 and GP5:pFc antigen could not be detected by ELISA.

|  | | | |
| --- | --- | --- | --- |
| **Antigen** | **Line** | **mg/g DS (+/- SD)** | **% of TSP** |
| GP3-Tm:pFc | Ho 5-1-9 | 2.905 +/- 0.289 | 0.96 +/- 0.09 |
| GP3-Tm:mFc2a | Ho 1-1-8 | 6.185 +/- 2.235 | 1.81 +/- 0.36 |
| GFP:GP3-Tm | Ho 5-3-2 | 7.858 +/- 0.404 | 2.74 +/- 0.16 |
| GP4 | Ho 5/1-5 | 0.245 +/- 0.033 | 0.08 +/- 0.01 |
| GP4:pFc | Ho 1-5-3 | 0.485 +/- 0.048 | 0.14 +/- 0.01 |
| GFP:GP4 | Se 1-2 | 0.467 +/- 0.038 | 0.15 +/- 0.02 |
| GP4-Tm | Ho 5/3-4-1 | 0.170 +/- 0.015 | 0.05 +/- 0.01 |
| GP4-Tm:mFc3 | Ho 3-5-13 | 0.151 +/- 0.019 | 0.05 +/- 0.01 |
| GP4-Tm:pFc | Ho 3-1-4 | 2.064 +/- 0.086 | 0.67 +/- 0.04 |
| GP4-Tm:mFc2a | Ho 3-1-4 | 5.153 +/- 0.276 | 1.59 +/- 0.11 |
| GFP:GP4-Tm | Ho 5-4-5 | 7.377 +/- 0.334 | 2.36 +/- 0.30 |
| sGP4-Tm | Se 3-6 | 0.019 +/- 0.003 | 0.005 +/- 0.001 |
| sGP4-Tm:pFc | Se 4-15 | 0.183 +/- 0.014 | 0.06 +/- 0.002 |
| sGP4-Tm:mFc2a | Ho 2-3-1-1 | 0.133 +/- 0.011 | 0.04 +/- 0.01 |
| GP5 | Ho 3/2-5-1 | *0.273* +/- - | *0.10* +/- - |
| GP5:pFc | Ho 5-1-3-2 | *0.015* +/- - | *0.01* +/- - |
| GP5-Tm | Ho 4/9-E-7 | 0.258 +/- 0.059 | 0.09 +/- 0.005 |
| GP5-Tm:pFc | Se 1-15 | 2.333 +/- 0.077 | 0.83 +/- 0.022 |
|  |  |  |  |
