# Supplementary material for: Boosting In Planta Production of Antigens Derived from the Porcine Reproductive and Respiratory Syndrome Virus (PRRSV) and Subsequent Evaluation of Their Immunogenicity
Source: PLoS One. 2014 Mar 10;9(3):e91386. doi: 10.1371/journal.pone.0091386 (PMC3948849; doi:10.1371/journal.pone.0091386)
Supplement: Table S2 — (Multistep) extension PCR data. Are given: the cloning project involved, the target of amplification, the forward and reverse primer used in each reaction, the number of consecutive extension PCRs performed for each project and the annealing temperature of the first five and last twenty cycles. The Phusion® High-Fidelity DNA Polymerase (Finnzymes) was used for all reactions according to the manufacturer's guidelines. Abbreviations used; T ann, annealing temperature. (DOCX) [file pone.0091386.s002.docx]

**Table S2. (Multistep) extension PCR data.** Are given, the cloning project involved, the target of amplification, the forward and reverse primer used in each reaction, the number of consecutive extension PCRs performed for each project and the annealing temperature of the first five and last twenty cycles. The Phusion® High-Fidelity DNA Polymerase (Finnzymes) was used for all reactions according to the manufacturer’s guidelines. Abbreviations used; T ann, annealing temperature.

|  |  |  | **Primer** | | **T ann** | |
| --- | --- | --- | --- | --- | --- | --- |
| **Project** | **Target of amplification** | **PCR reaction** | **Forward** | **Reverse** | **5x** | **20x** |
| Cloning GP3(-Tm):pFc | GP3(-Tm) | 1 | ropir1 | ropir12 | 65 | 72 |
|  |  | 2 | ropir1 | ropir4 | 64 | 69 |
|  |  | 3 | ropir1 | ropir5 | 61 | 72 |
| Cloning GP4(-Tm):pFc | GP4(-Tm) | 1 | ropir9 | ropir10 | 58 | 62 |
|  |  | 2 | ropir1 | ropir4 | 62 | 69 |
|  |  | 3 | ropir1 | ropir5 | 60 | 69 |
| Cloning sGP4(-Tm):pFc | sGP4(-Tm) | 1 | ropir1 | ropir2 | 54 | 71 |
|  |  | 2 | ropir1 | ropir5 | 60 | 69 |
| Cloning GP4:pFc | GP4 | 1 | ropir9 | ropir10 | 58 | 62 |
|  |  | 2 | ropir1 | ropir4 | 62 | 69 |
|  |  | 3 | ropir1 | ropir5 | 60 | 69 |
| Cloning GP5(-Tm):pFc | GP5(-Tm) | 1 | ropir9 | ropir10 | 58 | 62 |
|  |  | 2 | ropir1 | ropir4 | 62 | 69 |
|  |  | 3 | ropir1 | ropir5 | 60 | 69 |
| Cloning GP5:pFc | GP5 | 1 | ropir9 | ropir10 | 58 | 62 |
|  |  | 2 | ropir1 | ropir4 | 62 | 69 |
|  |  | 3 | ropir1 | ropir5 | 60 | 69 |
| Cloning GP4(-Tm):mFc3 | GP4(-Tm) | 1 | ropir1 | mFc3R3 | 52 | 70 |
| Cloning GP3(-Tm):mFc2a | GP3(-Tm) | 1 | ropir9 | mFc2aR2 | 54 | 63 |
| Cloning GP4(-Tm):mFc2a | GP4(-Tm) | 1 | ropir1 | mFc3R3 | 52 | 70 |
| Cloning sGP4(-Tm):mFc2a | sGP4(-Tm) | 1 | ropir9 | mFc3R3 | 52 | 63 |
| Cloning GFP:GP4(-Tm) | GFP:GP4(-Tm) | 1 | ropir1 | JaR5 | 53 | 69 |
|  |  | 2 | ropir1 | JaR6 | 65 | 69 |
| Cloning GFP:GP3(-Tm) | GP3(-Tm) | 1 | JaF7 | JaR8 | 58 | 71 |
