# Supplementary material for: Boosting In Planta Production of Antigens Derived from the Porcine Reproductive and Respiratory Syndrome Virus (PRRSV) and Subsequent Evaluation of Their Immunogenicity
Source: PLoS One. 2014 Mar 10;9(3):e91386. doi: 10.1371/journal.pone.0091386 (PMC3948849; doi:10.1371/journal.pone.0091386)
Supplement: Table S3 — Primer sequences. (DOCX) [file pone.0091386.s003.docx]

| **Table S3. Primer sequences** | |
| --- | --- |
| **Primer name** | **Sequence (5' to 3')** |
| ropir1 | CCGGAATTCCGGCCACCATGGCAAACAAGCTTTTCCT |
| ropir2 | AGATGGGTGTGGGGGGTTCGATGTCGCTTCCTCTTGGAACGAGGTGATGGTGATGGTGGTG |
| ropir4 | AGCAAATTTCGGGACAGATGGGTGTGGGGGGTTCGATGTCGCTTCCTCTTGGAACGAGGTGATGG |
| ropir5 | TACGCCTAGGACCTCTGCAGCTGGGCATGAGCAAATTTCGGGACAGATGGGTGT |
| ropir9 | TGAATTCCCACCATGGCAAACAAGCTTTTCCT |
| ropir10 | GCTTCCTCTTGGAACGAGGTGATGGTGATGGTGGTGGCTTGGTGTAAGGA |
| ropir12 | GCTTCCTCTTGGAACGAGGTGATGGTGATGGTGGTGTTCCAAGTGGAACC |
| mFc2aR2 | TGGACCCCGCGGTTCCAAGTGGAACCAGTTGCC |
| mFc3R3 | GTATCCGCGGGCTTGGTGTAAGGAAGTGGA |
| JaF7 | ATCACTCCGCGGACGAAGTTGATTCAAACTCCAGCTCTACGCTATGCTTT |
| JaR5 | TGGCGAACAAGCAAGCAATGGTTGTAGCCCACCTCATTGCGCTTGGTGTAAGGAAGTGGA |
| JaR6 | GGTGGATCCTTAAAGCTCGTCTTTGATTGCCAAGAGAATGGCGAACAAGCAAGCAATG |
| JaR8 | GGGTGGATCCTCAAAGCTCGTCTTTTTCCAAGTGGAACCAGTTGCCACCG |
